# The Brain of the Domestic *Bos taurus*: Weight, Encephalization and Cerebellar Quotients, and Comparison with Other Domestic and Wild Cetartiodactyla

**DOI:** 10.1371/journal.pone.0154580

**Published:** 2016-04-29

**Authors:** Cristina Ballarin, Michele Povinelli, Alberto Granato, Mattia Panin, Livio Corain, Antonella Peruffo, Bruno Cozzi

**Affiliations:** 1 Department of Comparative Biomedicine and Food Science, University of Padova, Viale dell’Università 16, 35020 Legnaro (PD), Italy; 2 Department of Psychology, Catholic University of Rome, Largo Gemelli 1, 20100 Milan (MI), Italy; 3 Department of Management and Engineering, University of Padova, Stradella S. Nicola 3, 36100 Vicenza (VI), Italy; University of Naples, ITALY

## Abstract

The domestic bovine *Bos taurus* is raised worldwide for meat and milk production, or even for field work. However the functional anatomy of its central nervous system has received limited attention and most of the reported data in textbooks and reviews are derived from single specimens or relatively old literature. Here we report information on the brain of *Bos taurus* obtained by sampling 158 individuals, 150 of which at local abattoirs and 8 in the dissecting room, these latter subsequently formalin-fixed. Using body weight and fresh brain weight we calculated the Encephalization Quotient (EQ), and Cerebellar Quotient (CQ). Formalin-fixed brains sampled in the necropsy room were used to calculate the absolute and relative weight of the major components of the brain. The data that we obtained indicate that the domestic bovine *Bos taurus* possesses a large, convoluted brain, with a slightly lower weight than expected for an animal of its mass. Comparisons with other terrestrial and marine members of the order Cetartiodactyla suggested close similarity with other species with the same feeding adaptations, and with representative baleen whales. On the other hand differences with fish-hunting toothed whales suggest separate evolutionary pathways in brain evolution. Comparison with the other large domestic herbivore *Equus caballus* (belonging to the order Perissodactyla) indicates that *Bos taurus* underwent heavier selection of bodily traits, which is also possibly reflected in a comparatively lower EQ than in the horse. The data analyzed suggest that the brain of domestic bovine is potentially interesting for comparative neuroscience studies and may represents an alternative model to investigate neurodegeneration processes.

## Introduction

The domestic bovine *Bos taurus* (Linnaeus, 1758) is a very common domestic mammal raised for meat and milk production (and sometimes still for field-work) almost everywhere in the world, or at least where climate and environmental conditions allow it. So it is safe to state that bovine farming is an industry with different levels of sophistication and technology, depending on the local economy and market. Approximately 5,5 million bovines are currently raised in Italy for commercial reasons, and several millions are slaughtered every year for meat production (> 2,500,000 for 2014) [1]. However, in spite of the widespread diffusion of this species, data on the bovine brain are comparatively scarce. Treatises of veterinary anatomy [2, 3, 4, 5, 6, 7, 8, 9] report data on the volume and/or weight of the brain, but details on the source (and especially on the number of subjects analyzed) are generally missing. Specific investigations on the morphology of the central nervous system (CNS) of large herbivores mostly date back to over one hundred years ago, when several researchers analyzed the brains of domestic animals, alone or in comparison with the human brain [10]. Not much else has been added since then, and as stated in the modern comprehensive textbook of [7] the identification of lobes in the brain of the bovine (and other domestic species) is currently impossible, due to the lack of information on the specific organization of the parts.

Information on the brain mass of the bovine comes from the literature or from museum collections and is generally outdated as well as arguably heterogeneous. Data on brain weight may result from indirect measurements based on cranial volume, and values reported in textbooks are apparently based on a small numbers of animals [3, 4, 5, 6, 7]. No mention is made whether the reported brain weights refer to fresh or formalin-fixed specimens. Therefore, data consistency becomes controversial when comparing different datasets, thus making results and possible correlations difficult to interpret.

Modern technologies, including magnetic resonance (MR), has allowed *in vivo* imaging of the bovine brain [11, 12] and comparison with other domestic species. It is now accepted that terrestrial Cetartiodactyla exhibit a highly folded cerebral cortex, but relatively low neuronal density [13]. The topographical identification of sulci and gyri is based on morphological analogies with the human and other well-known species, but the dearth of published reports on the cytoarchitecture of terrestrial Cetartiodactyla [14] makes it hard to establish functional correlations in the bovine. Since the cytoarchitectonics, neurochemistry, and connectivity of the cortical column is still poorly understood, identification of functions remains hypothetical and leaves several questions open [15].

It must be noted that, in the last decade, a number of studies have validated the use of *Bos taurus* species as interesting alternative mammalian species in comparative neuroscience studies, due to its large and highly convoluted encephalon and the length of the gestation period (41 weeks), that is comparable to the human pregnancy (38–40 weeks); for details see [16, 17, 18].

Many recent works have drawn attention to the relationship between evolutionary changes in brain size and behavioral complexity (for a recent review see [19]). The relationship of brain size to body size was the basis of Dubois’ “index of cephalization” [20]. Dubois’ proposal for an equation was further developed by [21], to obtain what was then referred to as the encephalization quotient (EQ). Encephalization occurs when the actual brain size diverges from the expected brain size for an animal of a given mass. Thus, EQ represents how many times larger (or smaller) a species’ brain is in comparison with what would be expected for its body mass [22]. The difference from the expected value is used as predictor of an animal’s adaptive capabilities.

Great attention has been devoted to inter- and intra-order comparisons among different mammalian species, almost constantly referring to wild specimens as a paradigm. Although the rationale of studying only undomesticated animals is quite clear, it is also true that the brain weight used to plot the EQ of some species may have been that of specimens who died in zoos or animal parks, therefore somehow far from the ideal. We emphasize that the application of EQ to the human brain of course is an exception. The issue of domesticated mammals has been seldom explored, except in a few articles [23]. In this context here we focused our attention to the brain of the domestic bovine *Bos taurus*.

The present paper is aimed at studying very essential but still indefinite issues regarding the size of bovine brain compared to body weight. The widely accepted EQ [21] and cerebellar quotient (CQ) allow comparison with other mammalian species to determine evolutionary patterns in brain size across divergent groups of mammals. These quotients have been calculated and applied to several groups of mammals, and especially to wild species. Although this is important to understand the evolutionary patterns of diversification and specialization of mammals, the total lack of information on domestic species is hard to explain, given their economic and social importance, and the important ethical issues related to animal welfare. In the present study, data obtained from a large number of bovine brains collected at the slaughterhouse are compared to data on the brain size of other mammalian species gathered from the published literature.

## Materials and Methods

### Brain sampling

For the present study, we sampled a total of 158 bovine brains, 150 of which were collected at the “F.lli Tosetto”, abattoir of Campo San Martino, Padova, Italy (see Table 1 for body and brain weight; details of sampled animals are reported in S1 Dataset) and 8 were removed in the necropsy room of the Department of Comparative Biomedicine and Food Science of the University of Padova at Legnaro.

**Table 1:** Breeds of *Bos taurus* sampled at the slaughterhouse (n = 150).

Breed	Mean animal weight (Kg) and (Standard error of mean)	Mean brain weight (g) and (Standard error of mean)	N
Dairy cattle (Holstein-Friesian and other breeds with the same attitude)	583.92 (10.98)	476.91 (4.45)	105
Beef cattle (Charolaise-Limousine and other breeds with the same attitude)	578.99 (6.81)	479.39 (12.69)	11
Crossbred with double attitude	643.41 (19.81)	492.11 (9.67)	34
**Total**	**597.05 (9.30)**	**480.54** (**3.93)**	**150**

A The age of the animals was determined based on official documentation available at the moment of slaughtering (for the animals sampled at the abattoirs) or presented by the owners (for the animals sampled in the necropsy room). The large majority of slaughtered animals (n = 139) was represented either by heifers or cows, since male *Bos taurus* are generally not raised beyond puberty in the production system.

The cause of death of the bovines sampled in the necropsy room was related to fatal illnesses of various nature but not involving the CNS. All the animals whose brain was sampled in the necropsy room were adults (>3 years).

At the slaughterhouse, animals were treated according to the current European Community Council directive concerning animal welfare during the commercial slaughtering process, and constantly monitored under mandatory official veterinary medical care. The brains were weighed using a Bel Engineering S3201 precision scale (range 0.1 to 3,200 g). The dura mater was removed during extraction of the brain. The arachnoid was frequently broken during removal of the dura. The pia mater was generally left in place, since its careful elimination was impossible to perform at the slaughterhouse without risking damaging to the brains. Body weight was determined for each animal by the staff of the slaughterhouse.

Brains removed in the necropsy room (n = 8) were immersed in formalin for two weeks and stored at 4°C to allow hardening and proper fixation. Such a short-term immersion in formalin did not substantially modify brain weight [23]. The subdivision of the brain into its component parts was performed consistently by one of the Authors (BC), to avoid bias in subsequent sampling sessions. The spinal cord was transected from the brain at the level of the occipital foramen. The weight of the brain and its components derived from primary vesicles (telencephalon; diencephalon; mesencephalon; pons, cerebellum; myelencephalon) was calculated based on formalin-fixed brains after careful dissection.

### Encephalization Quotient and Cerebellar Quotient

The weight of the brain was related to body weight to obtain the EQ, calculated with the formula $EQ\;=\;\frac{E_{i}}{0.12P^{\frac{2}{3}}}$, where Ei and P are the mean weights of the brain and body, respectively [21]. We maintained the value of the exponent ($\frac{2}{3}≅0.67$) originally indicated by [21], although we are aware of alternative values for the slope (for review see [24]). The EQ in this study was calculated using only data from fresh brains (n = 150). The EQ was calculated for each adult bovine, using their specific brain and body weights. The data thus obtained were then compared to reports in the literature for other species (see below). When choosing the species for comparison, we deliberately included representative terrestrial and marine Cetartiodactyla.

To calculate the Cerebellar Quotient (CQ), we applied the formula $CQ=\frac{Cb_{vol}}{\left(0.145\;Mb^{0.978}\right)}$ proposed by [25], in which Cb_vol_ is the volume of the cerebellum (*Cb*_*vol*_ × 1.04 = *Cb*_*mass*_ × 0.96) [26], and M_b_ is the brain weight (= Ei).

### Statistical analysis

To investigate the relation between brain weight (BrW), as the response of our model, and a set of possible independent categorical and numerical predictors, such as sex (S), bovine breed (BB), age (A) and body weight (BoW), we performed an in-depth statistical analysis using the general linear model. Actually, we fitted the following ANCOVA-type (ANalysis of COvariance and VAriance) [27] linear model:

$$\text{ln}\left({BrW}_{ijk}\right)\;=\;\mu +\beta_{1j}S_{j}+\beta_{2k}{BB}_{k}+\beta_{3}A_{ijk}+\beta_{4}\text{ln}\left({BoW}_{ijk}\right)+\varepsilon_{ijk},$$

where ln(.) means the natural logarithm, *i* is the animal individual index, *j* is the two-level sex index, *k* is the bovine breed category index and ε_*ijk*_ are random terms assumed as independent and identically homoscedastic (with fixed variance) normal distributed random errors.

After removing/selecting the non-significant/significant predictors, by using a step-wise approach, we applied to our data a suitable empirical model, i.e., a response surface (or curve) model [27], where the terms surface or curve response models refer to the number of numerical predictors, i.e. only one (curve) or more than one (surface).

The statistical software Minitab (release Minitab 17.2.1) was employed for all analyses.

## Results

### Gross anatomy of the bovine brain

**Fig 1:**
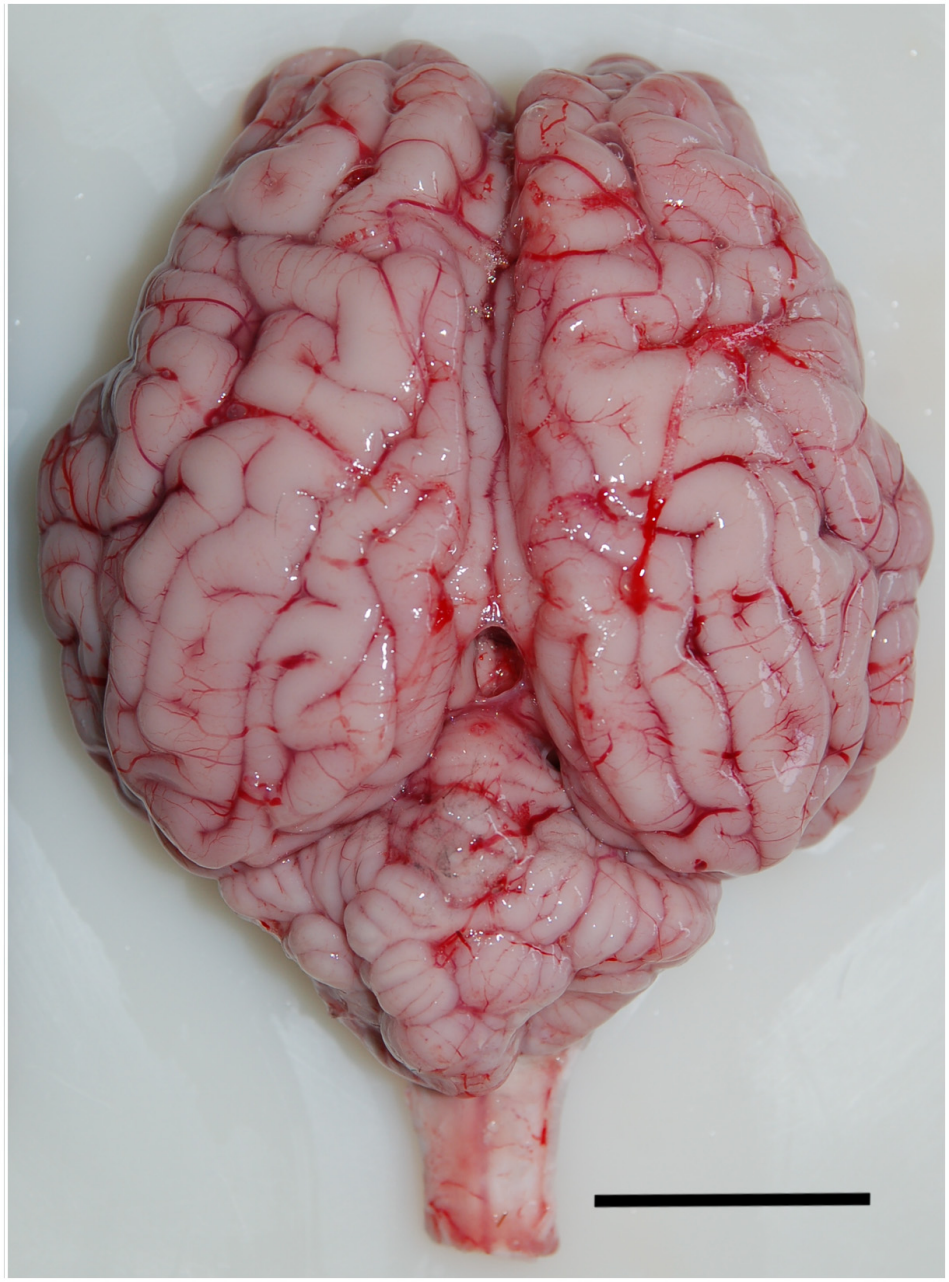
Dorsal view of the brain of a young *Bos taurus*. Scale bar = 3 cm.

The brains of the bovines of our experimental series exhibited gross morphological features typical of terrestrial Cetartiodactyla, with large telencephalic hemispheres, partially hidden cerebellum with prominent vermis, and an evident development of olfactory and limbic structures (Fig 1).

The small number of male specimens reflects the current production trend, in which only very few selected males are allowed to survive beyond puberty. Comparison between sexes was performed only for animals of comparable age (Table 2).

**Table 2:** Comparisons of young (1–2 years) male (n = 10) and female (n = 17) brains sampled at the slaughterhouse.

SEX	Mean animal weight (Kg) and (Standard error of mean)	Brain weight (g) and (Standard error of mean)	N
male	558.00 (33.68)	462.77 (11.14)	10
female	591.87 (24.84)	492.25 (9.85)	17

However, we emphasize that among the animals belonging to the same age class (1–2 years), females were generally a few months older than males and thus had heavier bodies and heavier brains. For substantiation of data see also the statistical analysis below.

### Weight of the brain and EQ

The mean brain weight of the bovines in our experimental series, based on a total of 150 animals and reported in Table 2, was 480.5 g (Standard error of mean—SEM = 3.9), with a mean body weight of 597.1 kg (SEM = 9.3). The mean weight of the brains fixed in formalin (n = 8) was 483.3 g (SEM = 21.33).

Table 3 reports body weight, brain weight and relative EQ values grouped according to the age class of the sampled animals.

**Table 3:** Age groups of the animals sampled at the slaughterhouse (n = 150).

Age group	N	Mean animal weight (Kg) and (Standard error of mean)	Brain weight (g) and (Standard error of mean)	EQ
1–3 years	40	580.95 (15.73)	476.19 (6.97)	0.58
4–8 years	94	603.66 (12.23)	480.41 (5.11)	0.57
≥ 9 years	16	598.44 (34.69)	492.14 (9.74)	0.59

The sample size is larger for animals in the 2–8 year range, as expected considering that the animals are raised for meat or milk production. The oldest cows were aged 16 (n = 1), and 15 (n = 4).

**Fig 2:**
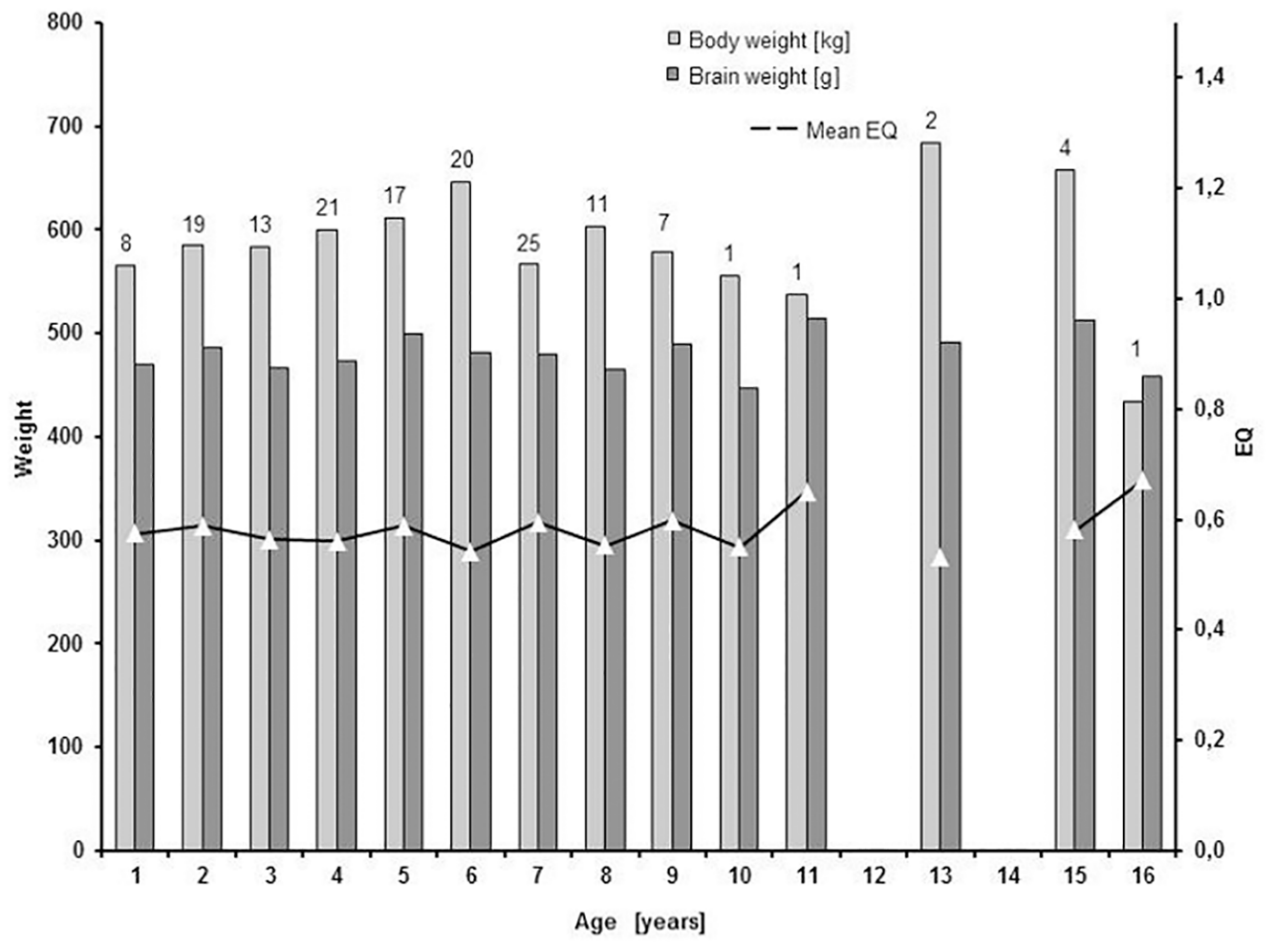
Body and brain weight of domestic *Bos taurus* in the different age classes. Light gray bars: body weight; dark gray bars: brain weight; solid line and white triangles: mean EQ. Numbers on top of the bars represent the number of specimens for each age considered.

Young pre-pubertal animals of less than three years (n = 40) had a brain weight of 476.19 g (SEM = 6.97), a mean body weight of 580.95 kg (SEM = 15.73), and an EQ of 0.58 (Fig 2 and Table 3). Bovine aged 4–8 years (n = 94) showed heavier brains (480.41 g, SEM = 5.11), heavier bodies (603.66 kg, SEM = 12.23) and a slightly lower EQ (0.57). The group of older animals aged 9–16 (n = 16) showed the highest EQ (0.59), due to heavier brains (492.14 g, SEM = 9.74) and a body weight intermediate between those of the former two age classes (598.44 kg, SEM = 34.69). Subdivisions of brain and body weight, and EQ, for single years of ages and age classes are represented in Fig 2.

The mean EQ, calculated using the mean values for brain and body weight of all animals (n = 150), was 0.565.

### Weight of brain vesicles and CQ

The absolute weights and relative percentages of the telencephalon, diencephalon, mesencephalon, cerebellum, pons, and medulla oblongata are summarized in Table 4.

**Table 4:** Absolute and relative weights of the constituents of the encephalon of the animals sampled in the necropsy room (n = 8).

Brain vesicle	Mean weight (g) and (Standard error of mean)	relative weight
Telencephalon	365.3 (16.21)	75.6%
Diencephalon	28.71 (1.61)	5.93%
Cerebellum	48.06 (2.25)	9.9%
Pons	13.86 (0.93)	2.9%
Medulla oblongata	18.47 (0.90)	3.8%
Whole brain	483.33 (21.33)	100%

The CQ calculated using mean cerebral and cerebellar masses of brains removed in the necropsy room (n = 8) was 0.725.

### Statistical results

We applied an ANCOVA-type general linear model by setting as response variable the logarithm of brain weight and as the set of possible independent predictors, two categorical variables such as sex, bovine breed, and two numerical variables such as age and logarithm of body weight. We also included some possible quadratic and interaction effects in our model, i.e. the square of age and the interaction between age and gender. Interestingly, the stepwise selection method suggested that, at the significant level set to 5%, only the body weight had a significant effect (P-value = 0.001) while all the remaining main and interaction effects were non-significant (Age P-value = 0.907, Sex P-value = 0.595, Bovine breed P-value = 0.537, Age^2^ P-value = 0.418, Age Gender P-value = 0.645). In particular, the insignificance role of the bovine age may be probably explained by the relative low number of young animals in our dataset. We remind that the stepwise selection method jointly removes and adds terms to the model for the purpose of identifying a useful subset of the terms.

**Fig 3:**
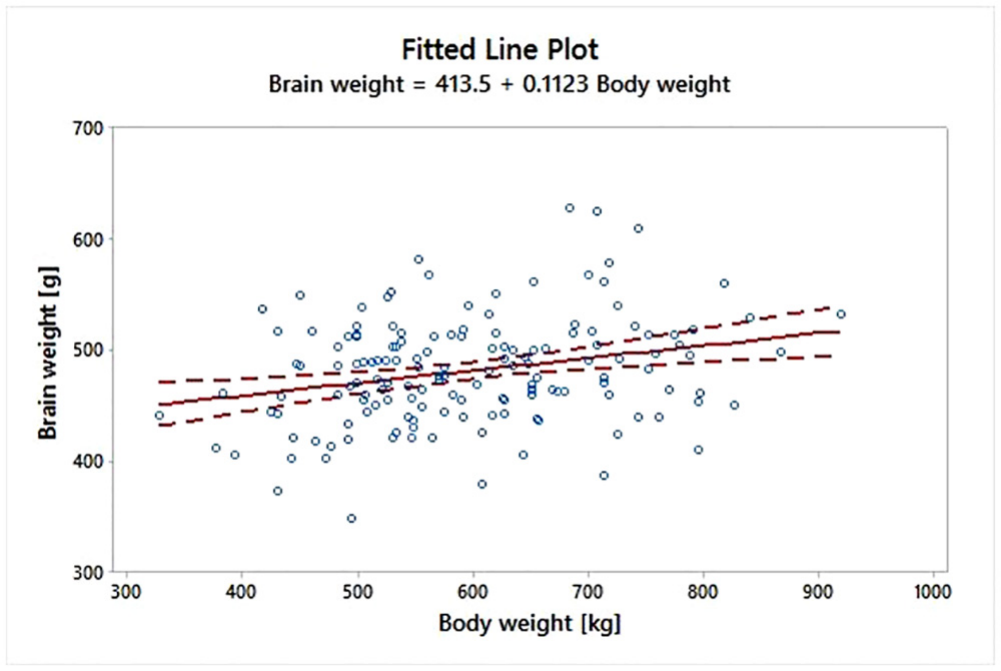
Linear regression analysis between logarithm of brain weight (g) *vs*. logarithm of body weight (kg). The fitted model was significant at the 5% significant level (P-value = 0.001).

After that, we fitted an Ordinary least squares (OLS) linear regression model (Fig 3) suitable to predict the logarithm of brain weight once setting the logarithm of body weight; the fitted model was significant at the 5% significant level (P-value = 0.001). Note that the empirical model was actually a linear curve since only one numerical predictors was found to significantly affect the brain weight.

**Fig 4:**
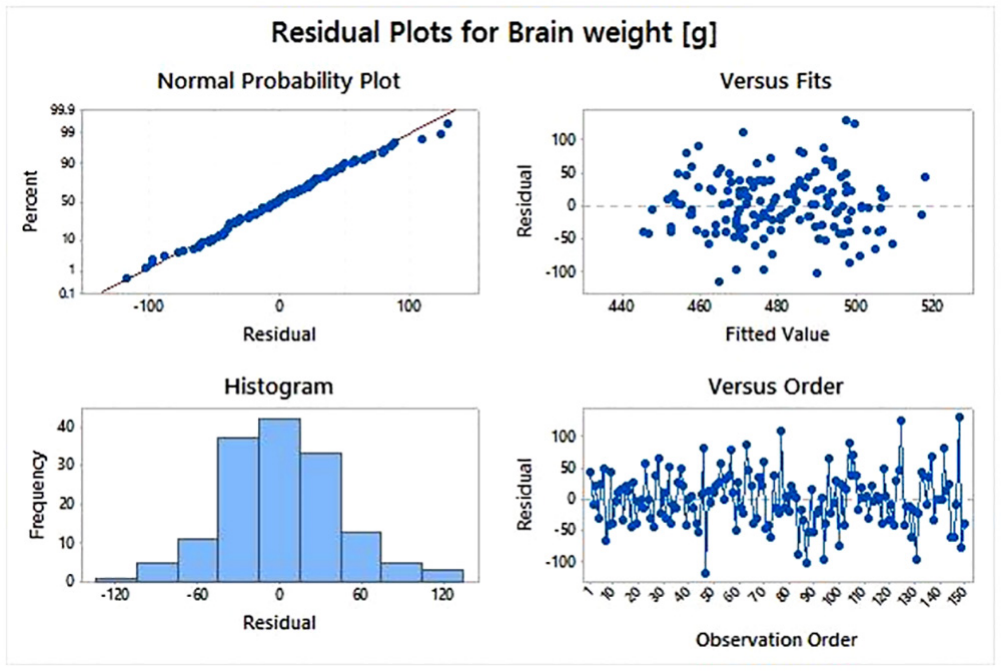
Graphical output from analysis of residuals of the linear regression model. The two left-side plots refer to the normality assumption while the two right-side plots refer to the homoscedasticity and independence assumptions.

The final residual analysis (Fig 4), suggested that the assumptions underlying the general linear model, and linear regression as well, i.e., normality, homoscedasticity, and independence, are all reasonably met. In fact, the plots on the left side shown a pretty straight line (Normal Probability Plot) and a very well bell-shaped histogram; finally, since no kind of structured patterns were found in the plots on the right side, the residuals versus fits and versus order plots did suggest that assumptions of homoscedasticity and independence are both met. We remind that residuals are actually calculated as the difference between the observed and the estimated response values; residuals can be argued of being informative on the theoretical random terms we assumed in our linear model (see Statistical analysis section).

## Discussion

In our experimental cohort, as expected, the brain weight increases in average as well as the body weight, with a positive slope equal to 0.5260 (in the logarithmic scale). It is worth noting that, even if there is some relative scatter around the fitted line, the mean brain weight is estimated with a relative small uncertainty, as demonstrated by the narrow confidence interval bands (Fig 3, dotted lines). In fact, the estimated error standard deviation was 0.097 ln(g). Therefore our data indicate that the brain of domestic *Bos taurus* shows a relatively low degree of variability, with minimal variations among age classes (Table 3 and Fig 2). This is clearly a consequence of the high degree of standardization of bovine farming, in which individuals are actively selected for specific genetic characteristics. This statement is confirmed by the outcome of our statistical analysis, where we showed that neither age nor bovine breed significantly affected the brain weight. The EQ that we obtained is similar (but not identical) to values reported by other studies in the same species [6, 28] (see Table 5).

**Table 5:** Brain weight, body weight and EQ in terrestrial Cetartiodactyla and selected marine species belonging to the same order.

Cetartiodactyla		Species	Brain weight (g)	Body weight (kg)	Reference	EQ
**terrestrial**	**Suidae**	*Sus scrofa*	180	192	[28]	0.45
			180	125	[29]	0.60
			162	157	[30]	0.46
	**Bovidae**	*Bos taurus*	445	550	[6]	0.55
			456	520	[28]	0.59
		*Ovis aries*	130	50	[6]	0.80
			125	49	[28]	0.78
			135	74	[29]	0.64
			137	46	[3]	0.89
			130	58	[7]	0.72
		*Capra hircus*	106	30	[28]	0.91
			125	38	[3]	0.92
			140	48	[7]	0.88
	**Camelidae**	*Camelus bactrianus*	576	400	[29]	0.88
			540	450	[30]	0.77
			518	594	[31, 32]	0.61
	**Giraffidae**	*Giraffa camelopardalis*	773	1002	[30]	0.64
			700	1209	[33]	0.51
**amphibious**	**Hippopotamidae**	*Hippopotamus amphibius*	882	2001	[34]	0.41
			590	1400	[28]	0.39
			720	1351	[33]	0.49
**aquatic**	**Delphinidae**	*Tursiops truncatus*	1587	167	[35]	4.4
			1676	215	[36]	3.89
			1759	206	[29]	4.20
		*Orcinus orca*	5617	2049	[35]	2.9
	**Physeteridae**	*Physeter macrocephalus*	7818	37094	[35]	0.6
	**Balaenopteridae**	*Megaptera novaeangliae*	6100	30050	[37]	0.53
			6439	39331	[38]	0.46

**Fig 5:**
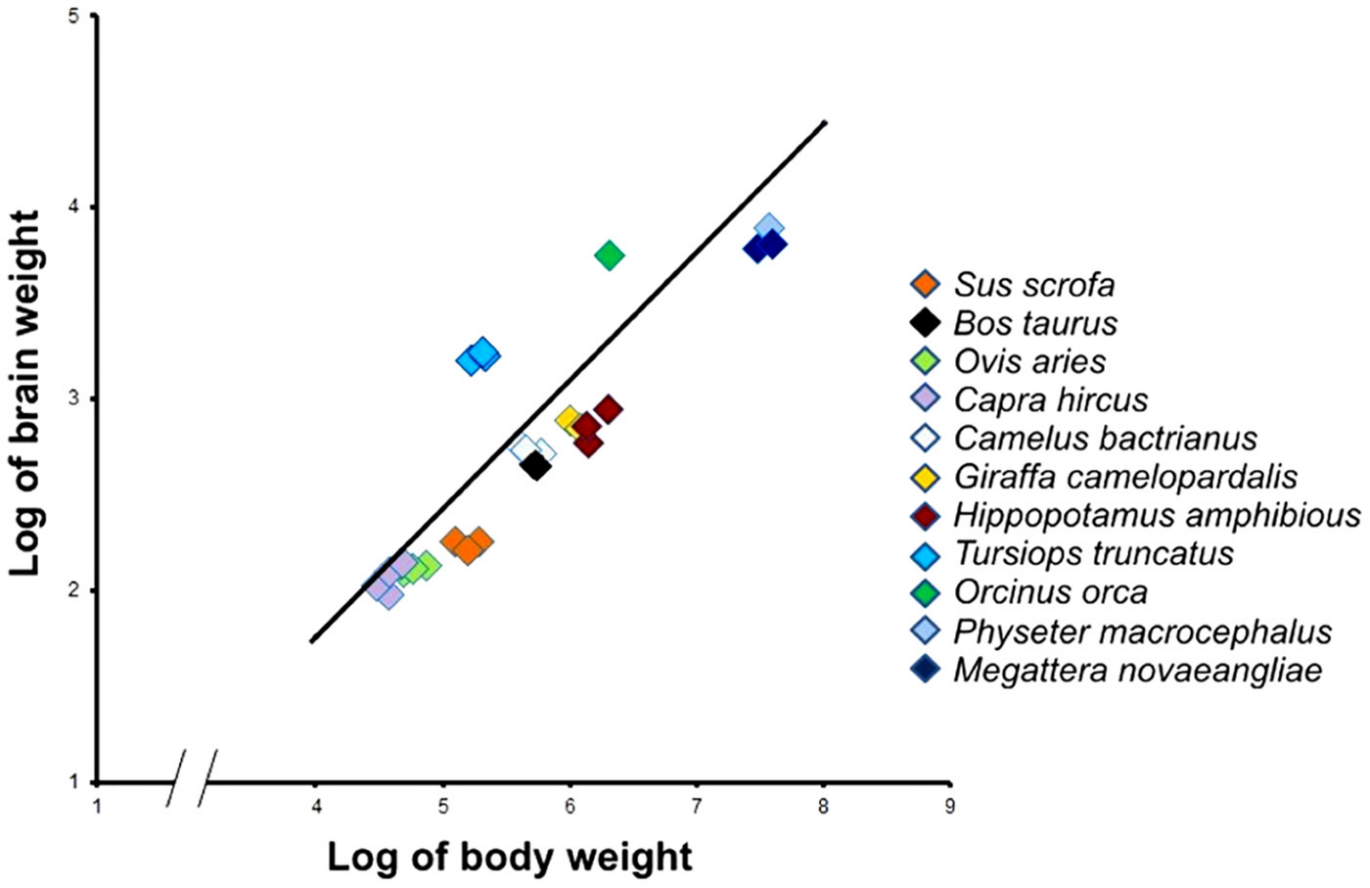
Encephalization Quotient (EQ) of terrestrial Cetartiodactyla and selected marine species belonging to the same order, based on brain and body weight expressed in grams.

The bovine EQ is also similar to that of other large terrestrial Cetartiodactyla, such as the Bactrian camel and the giraffe (Table 5 and Fig 5). The brain of the domestic *Bos taurus*, *Ovis aries* and *Capra hircus* are morphologically very similar [12], except for their mass. However, the sheep and goat present a higher EQ, possibly due to some degree of unavoidable bias of the Jerison’s equation towards species with high body mass (see also the hippopotamus). It is also remarkable that genetic selection of the domestic *Bos taurus* is far more advanced than that of other domestic herbivores, and obviously not comparable to wild species. There are presently no indications of possible differences in brain anatomy or volume due to a specific breed of domestic *Bos taurus*. The general description of the brain of this species given in classic textbooks of comparative neurology still applies [39]. Data on the swine are difficult to discuss and compare, because the reference studies reported in Table 5 did not detail the breed of the sampled individuals. We are presently investigating the EQ of the domestic *Sus scrofa* using a wide sampling cohort.

The EQ values of the bovine brain are similar to those of the large baleen whales, also belonging to the order Cetartiodactyla, and perhaps also emphasize the similarities in the feeding and digestive mechanisms shared by ruminants and baleen whales [40].

We emphasize that the situation is very different in the horse *Equus caballus*, another large and very common domestic herbivore belonging to the order Perissodactyla: The horse shows great individual variability n brain size, with approximately 10% of the animals showing an EQ in the primate range [23]. The difference between the even-toed bovine, and the odd-toed horse, species belonging to different mammalian orders, may depend on the less intensive breeding effort in horse farming. As is the case for other species [21], domestication of *Bos taurus* may have resulted in a loss of brain weight [41]. The evolutionary significance of the phenomenon is debated, and it appears that domestication has not affected the general dependency of brain on body size or vice versa, but domestication has changed the brain size [41]. It is also well known that no domesticated form has ever shown an increase in brain size in comparison to its wild counterpart [41].

The CQ (0.725) calculated for the animals sampled in the dissecting room (n = 8) was lower than that of the horse (0.841, [23]). When compared with other mammals, the bovine CQ was lower than that of African (>1.66), and Asian elephants (> 1.84); and primates (0.71–1.28) [25].

Attempts to correlate complex behaviors with brain structure or size are challenging (for a review see [42]). Differences in EQ and CQ between small toothed cetaceans and *Bos taurus* (and similar terrestrial Cetartiodactyla) are possibly related to evolutionary adaptation to different environments, feeding strategies and consequent sensorial specialization. However, the evolution of brain pathways that promote complex behavioral traits remains enigmatic [43]. Sensory centers in the brain increase in size and complexity in proportion to the importance of a particular sensory modality, yet often share circuit architecture because of constraints in processing sensory inputs [44]. Many different functional areas controlling sensitivity, motility or cognition are present within the brain and the evolution of these areas may occur according to two hypotheses. Specific areas may evolve independently [from other parts], according to the hypothesis of mosaic evolution [45]. Alternatively, a coordinated size change may occur, whereby functionally unrelated areas change together, as a result of the whole brain development [46, 47]. The fact that the bovine brain shows the lateral enlargement of the temporal lobes that is also typical of baleen whales (and elephants) may be due in part to a common genetic ancestry and to the need for high level acoustic performance. How the organization of the cortical column is affected by the process remains to be explained. The distribution of neural markers in the cortex is relatively well known in several orders of mammals, but not in terrestrial Cetartiodactyla [48]. Thus we may consider areas of the bovine brain (frontal, visual, cingulate) only as a general topographical reference, but not as a functional unit with definite homogeneity (for a general discussion on the homology of brain areas see [49]). Cytoarchitectonic maps (starting with [50]) are also essential to define the extension of individual areas, including the putative associative areas, where higher brain functions take place—at least in part—also in non-primate mammals. At present, we have no idea on the "quality" of the pre-cruciate cortex of bovine, and are so at a loss when defining this area, its extension and the reciprocal relationships with intra-cortical bundles and thalamic afferents. Indirect references [51] point to a diffuse lack of an evident layer 4 in the cortical columns, and thus suggest the presence of diffuse agranular microcircuitry, based on the analogue cortex observed in rodents [52] and man [53].

An alternative approach to comparative neuroscience, based on non-rodent models, may yield novel insights into the evolution of the brain and intelligence [54, 55]. A study of the neocortex in terrestrial Cetartiodactyla would be highly helpful for this purpose. Standardization of bodily parameters sets the domestic *Bos taurus* apart from its wild varieties (*Bos primigenius*), and may consequently diminish the general scientific interest towards this mammals. However, standardization is also a key requisite that guarantees reproducible results in scientific experiments, considering also that the use of bovine fetal and adult brain samples may contribute to reduce the use of laboratory animals, as unanimously proposed by several neuroscience societies and by the European Community regulations [56]. On the other hand, a better understanding of the neural abilities and sensory modalities of farm animals is also becoming crucial in view of the growing public concerns for their health and wellbeing, and the increasing awareness of their conditions.

## Supporting Information

S1 Dataset Details on the animal sampled at the slaughterhouse and in the necropsy room. The excel columns contain details on the single individual animals sampled at the slaughterhouse, including ear tag, brain weight, body weight, and breed.
(XLS)

## References

[1] National Register for Bovine (BDN), Ministry of Health and Institute for Animal Health “G.Caporale”, Teramo, Italy. Available http://issuu.com/istitutog.caporale/docs/annuario_bovino_dicembre_2013/0.

[2] Chauveu A, Arloing S. Traité d’anatomie comparée des animaux domestiques, 3rd ed Baillière, Paris, 1879, pp 1–1036.

[3] Zimmerl U. Il sistema nervoso In: Bossi V, Caradonna GB, Spampani G, Varaldi L, Zimmerl U (editors), Trattato di Anatomia veterinaria, Vol 3, Vallardi, Milano, 1909, pp 4–294.

[4] Ellenberger W, Baum H. Handbuch der vergleichenden Anatomie der Haustiere, 13th ed A. Hirschwald Publisher, Berlin, 1912, pp. 1–1104.

[5] Sisson S. The anatomy of the domestic animals. W.B. Saunders Co, Philadelphia, 1914, pp. 1–942.

[6] Seiferle E. Sistema nervoso, ghiandole endocrine, organi di senso In: Nickel R, Schummer A, Seiferle E (editors), Trattato di anatomia degli animali domestici. Vol. 4, Casa Editrice Ambrosiana, Milano, 1988, pp. 1–440.

[7] Barone R, Bortolami R. Anatomie comparée des mammifères domestiques. Tome 6, Neurologie I, Système Nerveux Central. Editions Vigot, Paris, 2004, pp.1–652.

[8] König HE, and Liebich H-G. Veterinary anatomy of domestic mammals. 4th ed Schattauer Verlag, Stuttgart, Germany, 2009, pp. 1–787.

[9] Dyce KM, Sack WO, Wensing CJG. Veterinary Anatomy, 4 ed, Elsevier, Amsterdam-New York, 2010, pp. 1–834.

[10] Tenchini L, Negrini F. Sulla corteccia cerebrale degli equini e dei bovini studiata nelle sue omologie con quella dell'uomo. Battei, Parma; 1889.

[11] Schmidt MJ, Pilatus U, Wigger A, Kramer M, Oelschlager A. Neuroanatomy of the calf brain as revealed by high-resolution magnetic resonance imaging. J Morphol. 2009; 270: 745–758. 10.1002/jmor.10717 19123244

[12] Schmidt MJ, Langen N, Klumpp S, Nasirimanesh F, Shirvanchi P, Ondreka N, Kramer M. A study of the comparative anatomy of the brain of domestic ruminants using magnetic resonance imaging. Vet J. 2012; 191: 85–93. 10.1016/j.tvjl.2010.12.026 21277239

[13] Kazu RS, Maldonado J, Mota B, Manger PR, Herculano-Houzel S. Cellular scaling rules for the brain of Artiodactyla include a highly folded cortex with few neurons. Front Neuroanat. 2014; 8:128 10.3389/fnana.2014.00128 25429261 PMC4228855

[14] Butti C, Raghanti MA, Sherwood CC, Hof PR. The neocortex of cetaceans: cytoarchitecture and comparison with other aquatic and terrestrial species. Ann N Y Acad Sci. 2011; 1225: 47–58. 10.1111/j.1749-6632.2011.05980.x 21534992

[15] Nieuwenhuys R, ten Donkelaar HJ, Nicholson C. The central nervous system of vertebrates, Vol 3, Springer-Verlag, Heidelberg; 1998 p. 1640.

[16] Peruffo A, Giacomello M, Montelli S, Corain L, Cozzi B. Expression and localization of aromatase P450AROM, estrogen receptor-α, and estrogen receptor-β in the developing fetal bovine frontal cortex. Gen Comp Endocr. 2011; 172: 211–217. 10.1016/j.ygcen.2011.03.005 21397601

[17] Panin M, Corain L, Montelli S, Cozzi B, Peruffo A. Gene expression profiles of estrogen receptors-α and-β in the fetal bovine hypothalamus and immunohistochemical characterization during development. Cell Tissue Res. 2015; 359: 619–26. 10.1007/s00441-014-2023-5 25363750

[18] Montelli S, Suman M, Corain L, Cozzi B, Peruffo A. Sexually Diergic Trophic Effects of Estradiol Exposure on Developing Bovine Cerebellar Granule Cells. Neuroendocrinology. 2016 . 26882349 10.1159/000444528

[19] Roth G. Convergent evolution of complex brains and high intelligence. Phil Trans R Soc B 2015; 370: 20150049 10.1098/rstb.2015.0049 PMC4650126 26554042

[20] Dubois E. Sur le rapport du poids de l’encéphale avec la grandeur du corps chez le mammifères. Bull Soc Anthropol Paris 1897; 8: 337–376.

[21] Jerison HJ. Evolution of the brain and intelligence. Academic Press, New York and London; 1973 pp.1–482.

[22] Boddy AM, McGowen MR, Sherwood CC, Grossman LI, Goodman M, Wildman DE. Comparative analysis of encephalization in mammals reveals relaxed constraints on anthropoid primate and cetacean brain scaling. J Evol Biol. 2012; 25: 981–994 10.1111/j.1420-9101.2012.02491.x 22435703

[23] Cozzi B, Povinelli M, Ballarin C, Granato A. The brain of the horse: weight and cephalization quotients. Brain Behav Evol. 2014; 83: 9–16. 10.1159/000356527 24335261

[24] Kruska DCT. On the evolutionary significance of encephalization in some eutherian mammals: effects of adaptive radiation, domestication, and feralization. Brain Behav Evol. 2005; 65: 73–108. 10.1159/000082979 15627722

[25] Maseko BC, Spocter MA, Haagensen M, Manger PR. Elephants have relatively the largest cerebellum size of mammals. Anat Rec. 2012, 295: 661–672. 10.1002/ar.22425 22282440

[26] Weaver AH. Reciprocal evolution of the cerebellum and neocortex in fossil humans. Proc Natl Acad Sci USA 2005; 102: 3576–3580. 15731345 10.1073/pnas.0500692102 PMC553338

[27] Vik PW. Regression, ANOVA, and the General Linear Model: A Statistics Primer. SAGE Publications, Los Angeles; 2014 pp. 1–344.

[28] Sacher GA, Staffeldt EF. Relation of gestation time to brain weight for placental mammals: implications for the theory of vertebrate growth. American Naturalist 1974; 108: 593–615.

[29] Shultz S, Dunbar RIM. Encephalization is not a universal macroevolutionary phenomenon in mammals but is associated with sociality. Proc Natl Acad Sci USA 2010; 107:21582–21586. 10.1073/pnas.1005246107 21098277 PMC3003036

[30] Pérez-Barberia FJ, Gordon IJ. Gregariousness increases brain size in ungulates. Oecologia 2005; 145: 41–52. 16032436 10.1007/s00442-005-0067-7

[31] Xie Z-H, Sun S-G, She Q-S, Chen L-Y, Wang J. Stereological estimation of volumes and cortical surface areas of the cerebrum and cerebellum in fixed bactrian camel (*Camelus bactrianus*) brain In: Gaholt TK, Saber AS, Nagpal SK, Wang J, editors. Selected research on gross anatomy and histology of camels. Camel Publishing House, Bikaner, 2011a pp 355–360.

[32] Xie Z-H, Li H-Y, Wang J (2011b) Morphological study of the cerebrum of bactrian camel (*Camelus bactrianus*) with particular reference to sulci In: Gaholt TK, Saber AS, Nagpal SK, Wang J, editors. Selected research on gross anatomy and histology of camels. Camel Publishing House, Bikaner, 2011b. pp 361–366.

[33] Silva M, Downing JA. The allometric scaling of density and body mass: A non-linear relationship for terrestrial mammals. American Naturalist 1995; 145: 704–727.

[34] Weston EM, Lister AM. Insular dwarfism in hippos and a model for brain size reduction in *Homo floresiensis*. Nature 2009; 459: 85–88. 10.1038/nature07922 19424156 PMC2679980

[35] Ridgway SH. Dolphin brain size In: Bryden MM, Harrison R, editors, Research on Dolphins, Oxford University Press, New York, 1986 pp.59–70.

[36] Marino L. Brain Size Evolution In: Perrin WF, Wursig B, Thewissen JGM, editors., Encyclopedia of marine mammals. Academic Press, San Diego, 2002 pp. 158–162.

[37] Jacobs MS, Jensen AV. Gross aspects of the brain and a fiber analysis of cranial nerves in the great whale. J Comp Neurol. 1964, 123: 55–72. 14199268 10.1002/cne.901230107

[38] Manger PR. An examination of cetacean brain structure with a novel hypothesis correlating thermogenesis to the evolution of a big brain. Biol Rev. 2006; 81: 293–338. 16573845 10.1017/S1464793106007019

[39] Frauchiger E, Hofmann W. Die nervenkrankheiten des rindes. Huber Verlag, Bern; 1941 pp.1–361.

[40] Sanders JG, Beichman AC, Roman J, Scott JJ, Emerson D, McCarthy JJ, et al Baleen whales host a unique gut microbiome with similarities to both carnivores and herbivores. Nature Communication 2015; 6:8285 10.1038/ncomms9285 PMC4595633 26393325

[41] Kruska DCT. The effects of domestication on brain size In: Krubitzer L, Kaas J, editors. Evolution of the nervous systems. Vol. 3: The evolution of nervous systems in mammals. Elsevier, London, 2007 pp. 143–153.

[42] Healy SD, Rowe C. A critique of comparative studies of brain size. Proc R Soc B 2007; 274: 453–464. 10.1098/rspb.2006.3748 PMC1766390 17476764

[43] Chakraborty M, Jarvis ED. Brain evolution by brain pathway duplication. Phil Trans R Soc B 2015; 370: 20150056 10.1098/rstb.2015.0056 26554045 PMC4650129

[44] Farris SM. Evolution of brain elaboration. Phil Trans R Soc B 2015; 370: 20150054 10.1098/rstb.2015.0054 PMC4650128 26554044

[45] Barton RA, Harvey PH. Mosaic evolution of brain structure in mammals. Nature 2000; 405:1055–1058 (10.1038/35016580). 10890446

[46] Finlay BL, Darlington RB. Linked regularities in the development and evolution of mammalian brains. Science 1995; 268:1578–1584 10.1126/science.7777856 7777856

[47] Molnár Z, Kaas JH, De Carlos JA, Hevner RF, Lein E, Němec P. Evolution and development of the mammalian cerebral cortex. Brain Behav Evol. 2014; 83: 126–139. 10.1159/000357753 24776993 PMC4440552

[48] Hof PR, Glezer II, Nimchinsky EA, Erwin JM. Neurochemical and cellular specializations in the mammalian neocortex reflect phylogenetic relationships: evidence from primates, cetaceans, and artiodactyls. Brain Behav Evol. 2000; 55: 300–310. 10971015 10.1159/000006665

[49] Watakabe A. Comparative molecular neuroanatomy of mammalian neocortex: What can gene expression tell us about areas and layers? Develop Growth Differ. 2009; 51: 343–354. 10.1111/j.1440-169X.2008.01085.x 19222526

[50] Brodmann K. Localisation in the cerebral cortex; 1909. Transl. by Garey J., London, Smith-Gordon; 1994.

[51] Hof PR, Glezer II, Condé F, Flagg RA, Rubin MB, Nimchinsky EA, et al Cellular distribution of the calcium-binding proteins parvalbumin, calbindin, and calretinin in the neocortex of mammals: phylogenetic and developmental patterns. J Chem Neuroanat. 1999; 16: 77–116. 10223310 10.1016/s0891-0618(98)00065-9

[52] Beul SF, Hilgetag CC. Towards a “canonical” agranular cortical microcircuit. Front Neuroanat. 2015; 8: 165 10.3389/fnana.2014.00165 25642171 PMC4294159

[53] Godlove DC, Maier A, Woodman GF, Schall JD. Microcircuitry of agranular frontal cortex: testing the generality of the canonical cortical microcircuit. J Neurosci. 2014; 34 5355–5369. 10.1523/JNEUROSCI.5127-13.2014 24719113 PMC3983808

[54] Manger PR, Cort J, Ebrahim N, Goodman A, Henning J, Karolia M, et al Is 21st century neuroscience too focussed on the rat/mouse model of brain function and dysfunction? Front Neuroanat. 2008; 2:5 10.3389/neuro.05.005.2008 19127284 PMC2605402

[55] Bolker J. There’s more to life than rats and flies. Nature 2012; 491: 31–33. 23128209 10.1038/491031a

[56] Peruffo A, Cozzi B. Bovine brain: An in vitro translational model in developmental neuroscience and neurodegenerative research. Front Pediatr. 2014; 2:74 10.3389/fped.2014.00074 25072040 PMC4090595

